# Bacteria from the Amphibian Skin Inhibit the Growth of Phytopathogenic Fungi and Control Postharvest Rots

**DOI:** 10.1007/s00248-025-02611-3

**Published:** 2025-09-30

**Authors:** Adriana J. Gutiérrez-Pavón, Martina María Pereyra, Florencia Isabel Chacón, Elizabeth Monroy-Morales, Eria A. Rebollar, Julián Rafael Dib, Mario Serrano, Yordan J. Romero-Contreras

**Affiliations:** 1 Centro de Ciencias Genómicas, Universidad Nacional Autónoma de México, Cuernavaca, Morelos México; 2 Facultad de Ciencias, Universidad Nacional Autónoma de México, Ciudad Universitaria, Ciudad de Mexico, México; 3 Planta Piloto de Procesos Industriales Microbiológicos (PROIMI), Consejo Nacional de Investigaciones Científicas y Técnicas (CONICET), Tucumán, Argentina; 4 Instituto de Microbiología, Universidad Nacional de Tucumán, Tucumán, Argentina

**Keywords:** Phytopathogenic fungi, Biological control agents, Postharvest diseases, Fruits

## Abstract

Postharvest diseases caused by phytopathogenic fungi represent one of the main challenges in the agricultural industry, leading to significant losses in fruit production. Although chemical treatments have been widely used for the control of these pathogens, the emergence of resistant strains and concerns regarding food safety and environmental impact have driven the search for novel effective and eco-friendly alternatives, such as the use of biological control agents (BCAs). Previously, we demonstrated that bacteria isolated from frog skin inhibit the growth of the phytopathogenic fungus *Botrytis cinerea*. Based on these findings, in this study we aimed to investigate the biocontrol potential of three bacterial isolates obtained from the skin of the frog *Craugastor fitzingeri*. Dual culture assays showed that these bacteria strongly inhibited the mycelial growth of several postharvest fungal phytopathogens, including *Penicillium digitatum*, *P. italicum*, *Alternaria alternata*, *Aspergillus niger*, and *Alternaria solani*. This antagonistic activity was further confirmed through assays using bacterial filtrates (BFs) and volatile organic compounds (VOCs), effectively delaying or suppressing fungal development under in vitro conditions. Additionally, in vivo experiments on citrus fruits, tomato, and blueberry demonstrated that treatments with bacterial cell suspensions or BFs significantly reduced disease incidence caused by *P. digitatum*, *A. alternata*, and *B. cinerea*. However, no inhibitory effects were observed against *Geotrichum citri-aurantii*, *Fusarium* sp., *Fusarium oxysporum*, and *Phytophthora capsici*, suggesting a degree of specificity. Our findings highlight the potential of frog skin-associated bacteria as a novel source of BCAs for the sustainable management of postharvest diseases in fruits.

**Supplementary Information:**

The online version contains supplementary material available at 10.1007/s00248-025-02611-3.

## Introduction

Postharvest diseases represent one of the major challenges in the agricultural industry, causing significant losses in both quantity and quality of plant-derived products [1, 2]. Many of these losses are caused by pathogenic fungi that induce rot diseases [3]. The pathogens responsible for these diseases belong to various genera, including *Penicillium*, *Botrytis*, *Alternaria*, *Colletotrichum*, *Geotrichum*, *Monilinia*, *Rhizopus*, *Aspergillus*, *Fusarium*, *Gloeosporium*, and *Mucor* [4–7]. Furthermore, several fungi also produce mycotoxins that are toxic to humans and other vertebrates [8]. Current strategies to control these diseases often rely on synthetic agrochemicals [9–11]. However, their use can negatively affect the environment and human health, highlighting the need for new ecological alternatives that are effective in disease control and ensure the production of safe and high-quality products [12, 13].

Biological control agents (BCAs) have emerged as promising eco-friendly tools to combat postharvest diseases [14, 15]. The BCAs are microorganisms and/or their byproducts capable of counteracting pathogen growth by antagonistic mechanisms, including the production of antifungal compounds, competition for nutrients and space, or the induction of plant defense responses [16–18]. The application of these BCAs is most effective when applied preventively before infection occurs [1]. Bacteria of the genera *Bacillus* and *Pseudomonas*, for instance, have been shown to control postharvest fruit diseases caused by *B. cinerea*, *A. alternata*, and *P. digitatum* [19–24]. Nevertheless, in many cases, the commercial application of BCAs remains limited because of their low success rate, which may be due to formulation factors, adaptation issues, biocontrol agent survival, or their development during storage and transport of the fruit [25, 26].

BCAs can be isolated from various sources, including plants (e.g., leaves, flowers, or fruits), infection sites, or storage facilities [1, 27, 28]. Beyond plant-associated environments, amphibian skin has also been identified as a reservoir of protective microbiomes. Amphibians harbor bacterial communities capable of inhibiting the chytrid fungus *Batrachochytrium dendrobatidis* (Bd) [29]. This protective capacity of host-associated microbiomes, exemplified in amphibians, highlights their potential as a source of novel BCAs for combating plant pathogens. In a previous work characterizing bacterial isolates from the frog *Craugastor fitzingeri*, we identified C26G and C32I as members of the genus *Acinetobacter*, and C23F as belonging to the *Enterobacteraceae* based on 16S rRNA sequencing. These bacteria improve the development and growth of tomato (*Solanum lycopersicum*) plants, inhibit the development of the pathogenic fungus *B. cinerea*, and induce protection mechanisms in blueberry fruits against this pathogen [30–32].

In this study, we evaluated the in vitro inhibitory effect of these three bacteria on fungi of agronomic interest, including *P. digitatum*, *P. italicum*, *A. alternata*, *Geotrichum citri-aurantii*, *Diplodia natalensis*, *A. niger*, *Phytophthora capsici*, *Fusarium* sp., *F. oxysporum*, and *A. solani*. Furthermore, we determined whether exogenous application of the bacteria protects citrus fruits, blueberries (*Vaccinium corymbosum*), and tomatoes against the pathogens *P. digitatum*, *A. alternata*, and *B. cinerea*.

## Materials and Methods

### Antifungal Activity Assay of Amphibian Skin Bacteria Against Plant Pathogens

Bacteria from amphibian skin were previously described [33]. The C23F, C26G, and C32I bacteria isolates were grown in Luria-Bertani (LB) medium at 30 °C for 24 h to an optical density of 0.6 (OD_600nm_). Spore suspensions of the phytopathogenic fungi *P. digitatum*, *P. italicum*, *A. alternata*, *G. citri-aurantii*, *D. natalensis*, *A. niger*, *P. capsici*, *F. oxysporum*, *Fusarium* sp., *A. solani*, and *B. cinerea* were prepared as previously described [34] (Supplementary Table 1). For in vitro antifungal assays, 60-mm Petri dishes containing PDA (potato dextrose agar) medium were inoculated at one end with 10 µl of each bacterial suspension with an optical density OD_600nm_ of 0.6, and at the opposite end with 6 µl of each fungus (1 × 10^6^ spores/ml). Control plates were inoculated with the pathogen suspension alone. The plates were incubated in the dark at 24 °C for 7 days. The test was done in triplicate (*n* = 3). Mycelial growth area measurement was calculated using ImageJ2 software.

### Antifungal Activity Assay of Volatile Organic Compounds (VOCs) Against Fungal Pathogens

To evaluate the antifungal activity of the volatile organic compounds produced by bacteria strains C23F, C26G, and C32I, we followed the methodology described in Romero-Contreras, Gonzalez-Serrano [30]. For each fungus, a spore suspension (1 × 10^6^ spores/ml) was inoculated at the center of a PDA medium plate, while a cell suspension of strains C23F, C26G, and C32I, adjusted to an OD_600nm_ of 0.6, was spread on another plate containing LB medium. Both plates were placed face to face, hermetically sealed with parafilm tape, and incubated at 26 °C for 7 days in the dark. The test was done in triplicate (*n* = 3). The evaluation of the mycelial growth area was calculated using ImageJ2 software.

### Antifungal Activity Assays of the Bacterial Filtrates Against Pathogenic Fungi

To evaluate the antifungal activity of the compounds secreted by the bacteria C23F, C26G, and C32I, we used the methodology previously described in Romero-Contreras, Gonzalez-Serrano [30]. The bacteria were grown at 30 °C in the dark until reaching an optical density (OD₆₀₀) of 0.6 in LB medium. Each bacterial culture was then centrifuged at 12,500 rpm (BECKMAN J2-21) for 15 min, and the supernatant was filtered through a 0.22-µm Millipore membrane. The bacterial filtrates were adjusted to a concentration of 80% (v/v). For each fungus, 6 µl of a spore suspension (1 × 10^6^ spores/ml) was inoculated in the center of the plate. The antifungal activity was evaluated after 5 to 7 days. The test was done in triplicate (*n* = 3). The evaluation of the mycelial growth area was calculated using ImageJ2 software [https://imagej.net/software/imagej2/].

### In Vivo Biocontrol Test on Fruits

To evaluate the protection efficiency of C26G, C23F, and C32I bacteria in vivo, we used lemons (*Citrus limon* L. Burm), sweet oranges variety Westin (*Citrus sinensis*), tangerine oranges (*Citrus x tangerine*), grapefruits (*Citrus x paradisi*), blueberries (*Vaccinium corymbosum* L.), and tomatoes (*Solanum lycopersicum*) at the commercial maturity stage. The fruits were disinfected with 70% ethanol. Subsequently, a bacterial cell suspension of each strain was prepared in 250 ml of LB medium to an OD_600nm_ of 0.6 and centrifuged at 12,500 rpm (BECKMAN J2-21) for 15 min. The cell pellet was resuspended in 25 ml of saline solution (NaCl 9 g/l). For bacterial consortia analysis, dual and tripartite combinations were prepared at a 1:1 and 1:1:1 volume ratio. Monoculture supernatants were collected and filtered through a 0.22-µm Millipore filter to obtain bacterial filtrates (BF), which were then resuspended in physiological solution [NaCl 9 g/l], to reach an 80% (v/v) concentration. A 3-mm incision was made in the equatorial zone of each fruit and sprayed with 2.5 ml of each bacterial suspension (mono-, dual-, or tripartite) or the BFs. Saline solution (without bacteria) was used as a control. The treated fruits were incubated at 25 ± 1 °C and 95% relative humidity for 24 h. Following this, fungal infection of citrus, blueberries, and tomato was done by spraying 50 ml of a spore suspension (1 × 10^6^ spores/ml) of *P. digitatum*, *A. alternata*, and *B. cinerea*, respectively. The fruits were then incubated at 25 ± 1 °C for 5 to 7 days post-inoculation. Disease incidence was measured by counting healthy and infected fruits. Three independent biological experiments were conducted, each with three technical replicates, using 5 to 10 fruits per replicate [27].

## Results

### Frog Skin Bacteria Have Antagonistic Activity Against Pathogenic Fungi

**Fig. 1:**
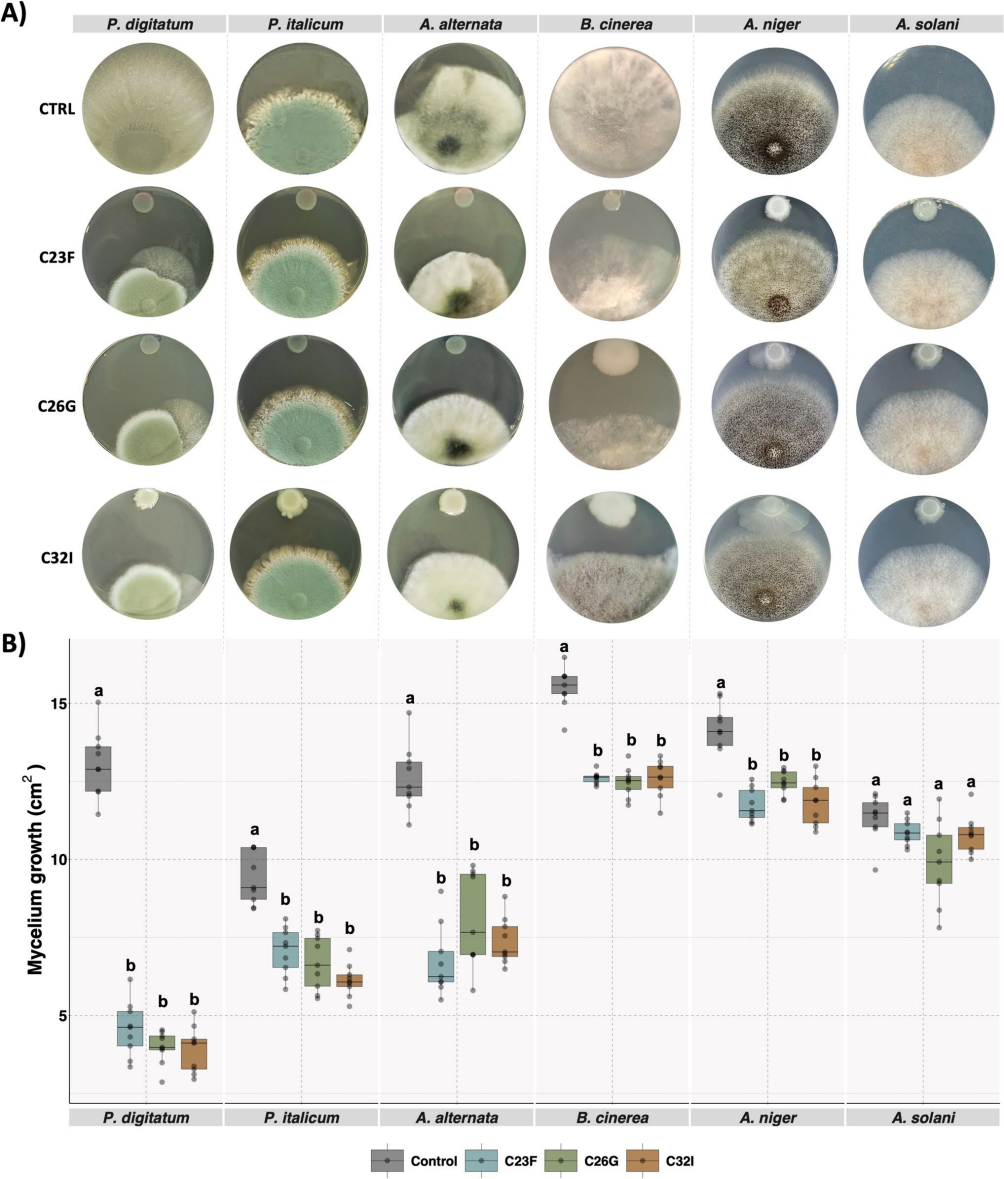
Inhibitory activity of bacteria from frog skin against fungal pathogens. Dual culture assays were performed to evaluate the interaction between the fungal pathogen and the bacterial isolates C23F, C26G, and C32I individually. Mycelial growth of each pathogen was assessed after 5 to 7 days of incubation to 27 °C. **A** Representative images showing the interaction between each bacterial isolate and the fungal pathogens *Penicillium digitatum*, *P. italicum*, *Alternaria alternata*, *Botrytis cinerea*, *Aspergillus niger*, and *A. solani*. **B** Quantitative assessment of mycelial growth for each pathogen. Data are presented as the mean of the standard deviation (± SD) of three biological replicates, each with three technical replicates. Different letters indicate statistically significant differences according to one-way ANOVA (*p* ≤ 0.05), followed by Tukey’s post hoc test

In previous studies, we demonstrated that bacteria isolated from frog skin inhibit the growth of the pathogenic fungus *B. cinerea* [30, 32]. Based on these findings, the bacteria C23F, C26G, and C32I were tested against various plant pathogenic fungi under in vitro conditions (Supplementary Table 1). The results showed that these bacteria inhibited the growth of *P. digitatum*, *P. italicum*, *A. alternata*,* and A. niger*, compared to the controls without bacteria (Fig. 1A). A mycelial growth analysis revealed that the C23F, C26G, and C32I bacterial strains differentially inhibited the growth of the tested pathogens. *P. digitatum* was the most affected, with growth radii of 4.5, 3.9, and 3.8 cm^2^, whereas *P. italicum* exhibited moderate inhibition of 7.0, 6.6, and 6.1 cm^2^. *A. alternata* showed intermediate sensitivity, with radii of 6.7, 8.0, and 7.3 cm^2^, while *A. niger* was the least affected, recording 11.0, 12.0, and 11.5 cm^2^, compared to control fruits that reached 13–15 cm^2^ (Fig. 1B). Conversely, no inhibitory effect was observed against *A. solani*, *F. oxysporum*, or *Fusarium* sp. (Fig. 1A, Supplementary Fig. 1).

### Volatile Organic Compounds Released by Amphibian Skin Bacteria Affected the Growth of Pathogenic Fungi

**Fig. 2:**
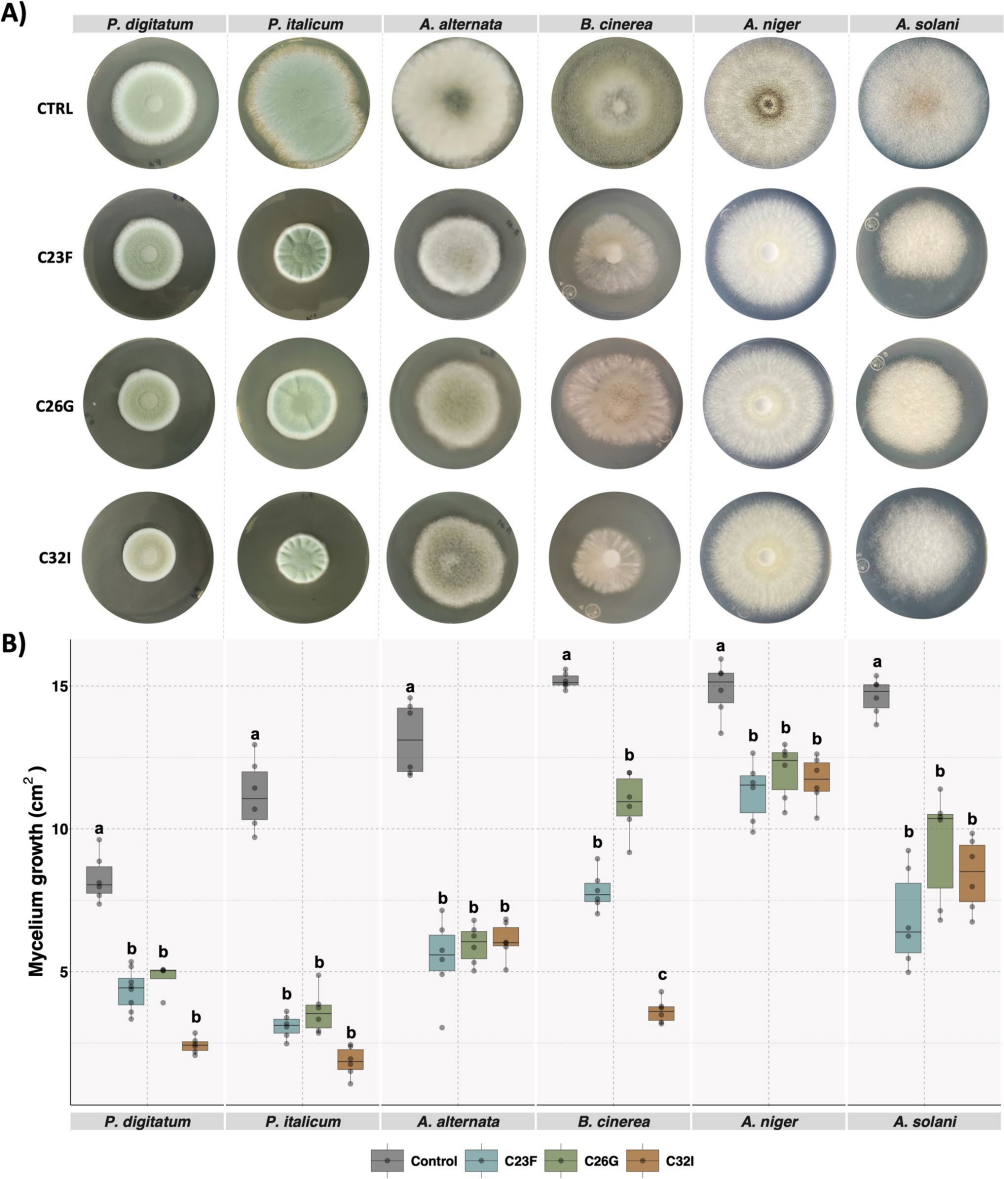
Compounds released by bacterial isolates C23F, C26G, and C32I delay the mycelial growth of fungal pathogens. A spore suspension of each fungal pathogen (4 × 10^4^ spores/ml) was placed at the center of a Petri dish containing PDA supplemented with 80% (v/v) bacterial filtrate (BF) from each strain and incubated at 24 °C for 5 to 7 days. **A** Representative images of the mycelial growth of each pathogen in the presence of the corresponding BF. **B** Fungal development was evaluated by measuring the mycelial area under each condition. PDA medium without BF was used as the control. Data are presented as the mean of the standard deviation (± SD) of three biological replicates, each with three technical replicates. Different letters indicate statistically significant differences according to one-way ANOVA (*p* ≤ 0.05), followed by Tukey’s post hoc test

Dual plate assays were performed to assess the antagonistic activity of VOCs, in which a bacterial culture plate was positioned inverted over a fungal culture plate. The bacterial strains C23F, C26G, and C32I inhibited the growth of six out of ten pathogenic fungi (Fig. 2A, Supplementary 2A). Analysis of fungal mycelial growth revealed that VOCs produced by C23F, C26G, and C32I strains differentially inhibited the pathogens. *P. digitatum* was the most susceptible, with a growth radii of 4.5, 4.7, and 2.4 cm^2^, followed by *P. italicum*, which exhibited moderate inhibition of 3.0, 3.5, and 1.8 cm^2^. *A. alternata* showed intermediate sensitivity, with growth radii of 5.4, 5.9, and 6.0 cm^2^, whereas *A. niger* was less affected, displaying 11.3, 12.0, and 11.6 cm^2^, and *A. solani* presented 6.8, 9.4, and 8.4 cm^2^, compared to the controls, which reached approximately 14 cm^2^ (Fig. 2B). In contrast, no inhibitory effect was observed against *G. citri-aurantii*, *F. oxysporum*, *Fusarium* sp., nor *P. capsici* (Supplementary Fig. 2B). These findings suggest that VOCs produced by bacterial strains C23F, C26G, and C32I have significant potential to inhibit important plant pathogens, particularly those responsible for postharvest diseases.

### Compounds Produced by the Bacterial Strains Inhibited the Growth of Pathogenic Fungi

**Fig. 3:**
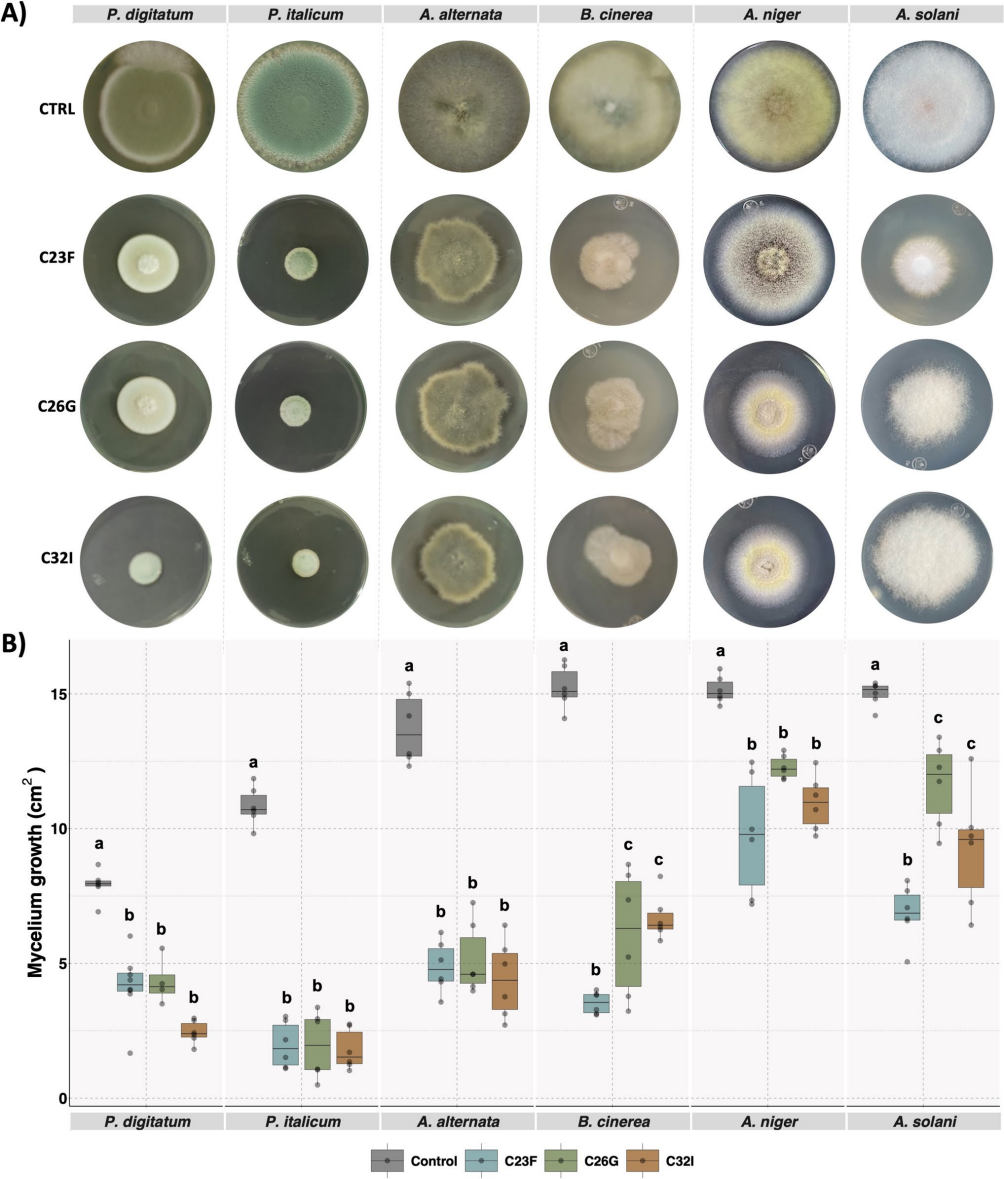
VOCs released by bacterial isolates C23F, C26G, and C32I contribute to the inhibition of fungal pathogens. Face-to-face (double plate) assays were performed to evaluate the inhibitory effect of volatile organic compounds (VOCs) produced by each bacterial strain on the growth of the fungal pathogens. **A** Representative images showing the inhibitory effect of bacterial VOCs on the fungal pathogens. **B** Analysis of the area of mycelial growth compared with the control. Data represent the mean (± SD) of three independent experiments, each performed in duplicate, and presented relative to controls. Letters indicate a statistically significant difference, according to one-way analysis of variance (ANOVA) (*p* ≤ 0.05) followed by the Tukey test

To evaluate the antifungal activity of the compounds released by strains C23F, C26G, and C32I, the bacterial filtrates (BFs) were tested against several pathogenic fungi. The inhibitory activity of BF was evaluated by measuring the mycelial growth area of the pathogens, with fungi grown without BFs used as controls. The results demonstrated that compounds released by C23F, C26G, and C32I in PDA medium significantly reduced the mycelial growth of several pathogens (Fig. 3A). *P. digitatum* exhibited growth radii of 4.3, 3.5, and 2.4 cm^2^ for the bacterial strains C23F, C26G, and C32I, respectively, followed by *P. italicum*, which showed moderate inhibition of 1.9, 2.0, and 1.7 cm^2^, while *A. alternata* displayed 4.8, 4.4, and 3.5 cm^2^, *A. niger* showed 9.7, 12.0, and 10.9 cm^2^, and *A. solani* recorded 6.8, 11.6, and 9.2 cm^2^ in the presence of the three bacterial strains, compared to the controls, which reached growth radii between 10 and 15 cm^2^ (Fig. 3B). In contrast, BFs did not affect the development of *G. citri-aurantii*, *Fusarium* sp., and *P. capsici*, as these exhibited growth rates that were comparable to their negative controls (Supplementary Fig. 3). In conclusion, the bacterial filtrates from strains C23F, C26G, and C32I demonstrated a strong inhibitory effect on the growth of certain pathogenic fungi, while other fungi showed lower sensitivity to these compounds.

### Frog Skin Bacteria Protect Lemons Against *P. digitatum*

**Fig. 4:**
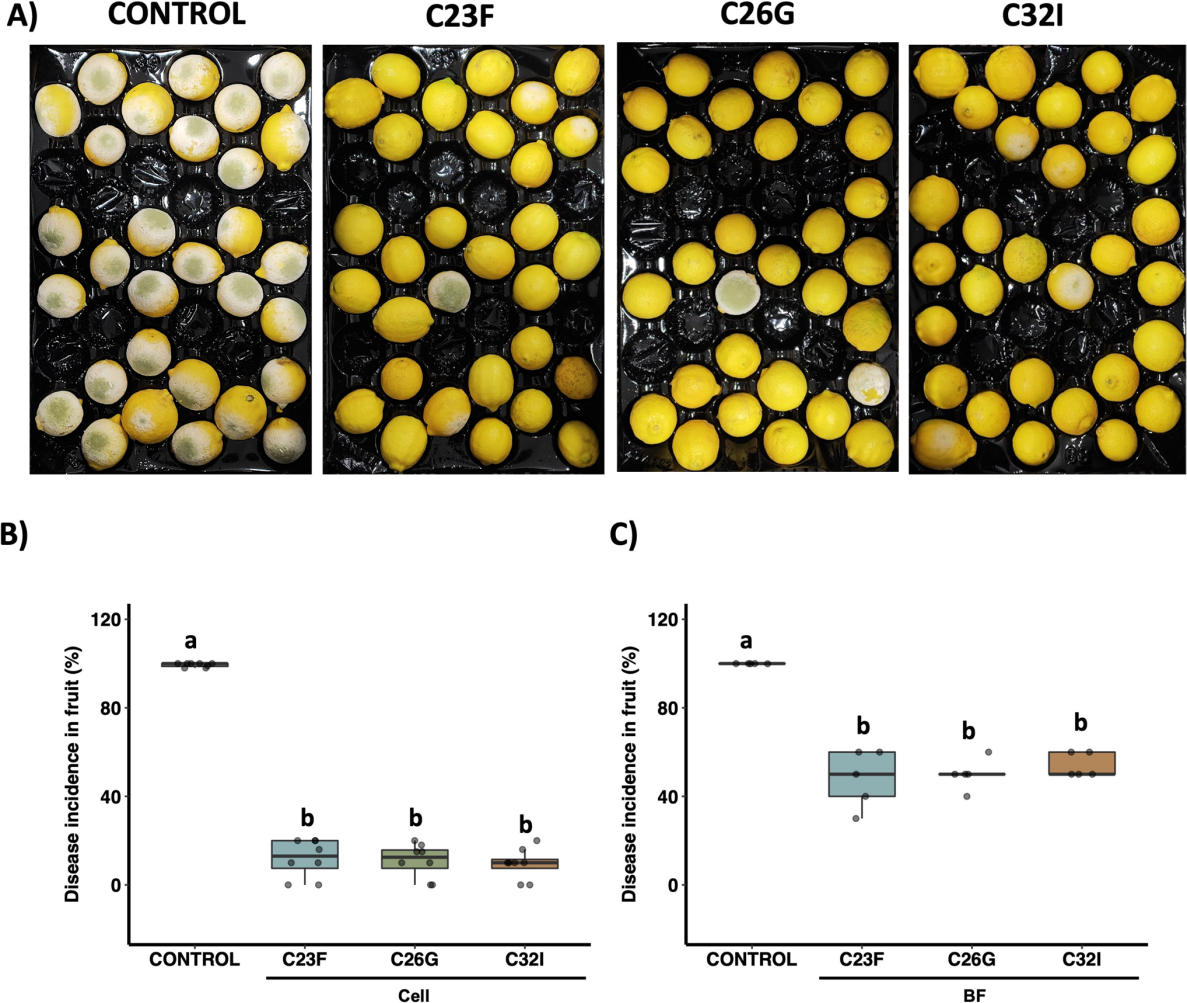
Biocontrol effect of bacterial isolates C23F, C26G, and C32I against fungal infection in lemon fruit. Lemons were wounded in the equatorial zone and treated with cell suspensions of bacterial isolates C23F, C26G, and C32I. Subsequently, fruits were inoculated with a spore suspension of *P. digitatum* and evaluated 5 days post-infection (dpi). **A** Representative images of the fruits under different treatment conditions. **B** Quantification of disease incidence in lemons treated with cell suspension. **C** Analysis of disease incidence in fruits treated with bacterial culture filtrates. Uninoculated lemons were used as controls. Different letters indicate statistically significant differences (*p* < 0.05). Three independent biological experiments were conducted, each with three technical replicates, using 10 fruits per replicate

To determine the effect of strains C23F, C26G, and C32I in protecting citrus fruits (*Citrus limon* L. Burm) against *P. digitatum* (green mold disease), in vivo tests were performed using cell suspensions and bacterial filtrates on wounded fruits. Lemons treated with each bacterial strain showed a lower incidence of disease compared to the control (Fig. 4A). Untreated lemons exhibited a disease incidence of 100%, while fruit treated with C23F, C26G, and C32I bacteria showed incidence of 12%, 11%, and 9.5%, respectively (Fig. 4B). In contrast, lemons treated with BFs showed incidences of 40%, 50%, and 54%, respectively (Fig. 4C). When the effect of these bacteria was evaluated on other citrus varieties, including Westin oranges (*Citrus sinensis*), tangerine oranges (*Citrus* × *tangerine*), and grapefruits (*Citrus paradisi*), a reduction in disease incidence was also observed (Supplementary Fig. 4). Furthermore, microbial consortia were also tested through dual and tripartite combinations of the bacterial strains. Lemons treated with the combinations C23F/C26G, C23F/C32I, and C23F/C26G/C32I exhibited disease incidences of 16%, 21%, and 16%, respectively. In contrast, the C26G/C32I combination was less effective, with a disease incidence of 41%. These results markedly differ from the control, in which 97% of the fruits developed disease symptoms (Supplementary Fig. 5). Overall, these results demonstrate the potential of these bacteria as biological control agents in the protection of citrus against green mold disease, suggesting that their effect can be effective across different citrus species.

### Frog Skin Bacteria Protect Tomato and Blueberry Fruits Against Fungal Pathogens

**Fig. 5:**
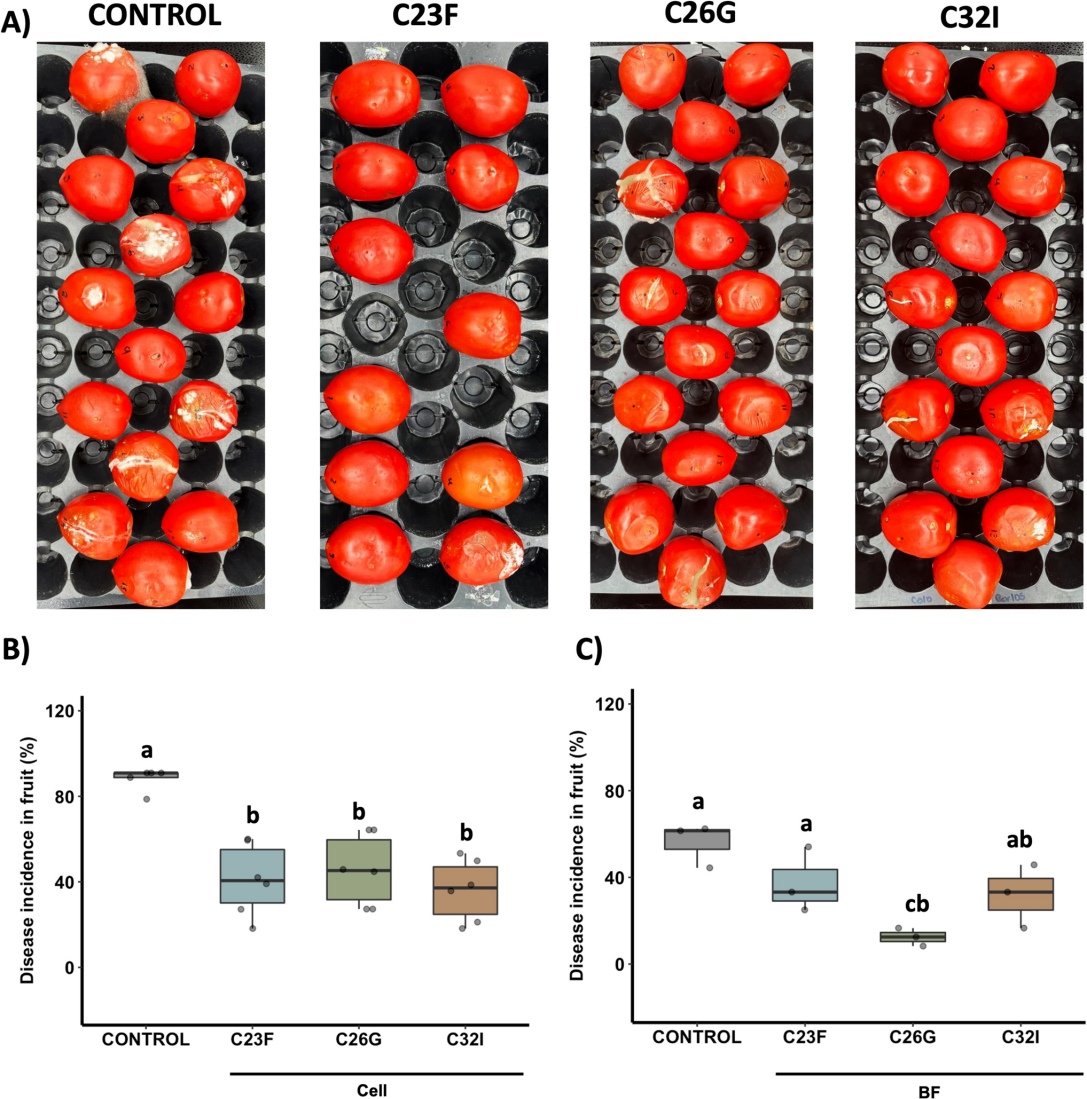
Bacterial isolates C23F, C26G, and C32I protect tomatoes from infection caused by *B. cinerea*. Tomatoes were wounded and treated with cell suspensions of frog skin bacteria C23F, C26G, and C32I. The fruits were then inoculated with a spore suspension of *B. cinerea* and incubated for 7 days at 24 °C. **A** Representative images showing the progression of infection in tomato fruits. **B**, **C** Analysis of disease incidence in tomatoes treated with bacterial cell suspensions and culture filtrates from each strain. Non-inoculated tomatoes were used as controls. Different letters indicate statistically significant differences (*p* < 0.05). Three independent biological experiments were conducted, each with three technical replicates, using 10 fruits per replicate

To evaluate the antagonistic activity of the bacterial strains C23F, C26G, and C32I in controlling gray mold (*B. cinerea*) in tomatoes, treatments with bacterial cell suspensions and filtrates were applied. We observed that both treatments significantly reduced disease development compared to the untreated control (Fig. 5). Tomatoes treated with any of the bacterial cell suspensions exhibited a 50% reduction in disease incidence, in contrast to the control, which showed an incidence of 92% (Fig. 5B). Treatment with bacterial filtrates reduced disease incidence to 37.4%, 12.4%, and 31.8% for the filtrates of C23F, C26G, and C32I, respectively (Fig. 5C).

**Fig. 6:**
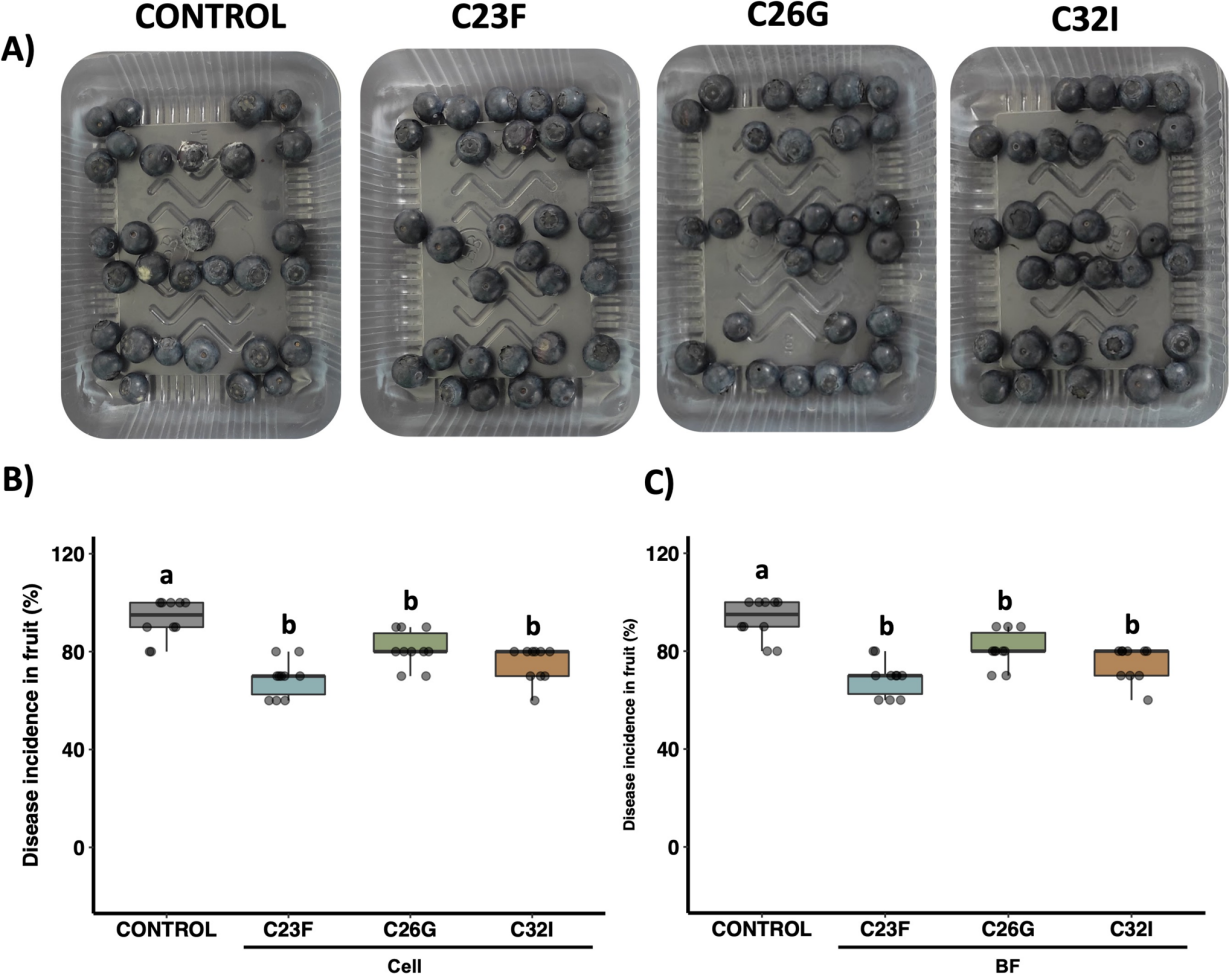
Frog skin bacteria inhibit *Alternaria alternata* in blueberry fruits. Blueberry fruits were treated with cell suspensions and bacterial culture filtrates from each strain and subsequently inoculated with *A. alternata*. Infection incidence was evaluated 7 days post-infection. **A** Representative images of infected fruits. **B**, **C** Analysis of disease incidence in fruits under each treatment and condition. Non-inoculated tomatoes were used as controls. Different letters indicate statistically significant differences (*p* < 0.05). Three independent biological experiments were conducted, each with three technical replicates, using 10 blueberries per replicate

In blueberries, both bacterial cell suspensions and filtrates from C23F, C26G, and C32I significantly reduced disease development (Fig. 6A). Treated fruits show a 70% reduction in disease incidence, compared to the control, which reached an infection of incidence of 98% (Fig. 6B, C). The results demonstrate that the evaluated bacterial strains possess a broad-spectrum protective effect against different phytopathogens across multiple crops.

## Discussion

Fruits are an essential component of the human diet and are important for human health due to their rich nutritional content, including vitamins, minerals, and antioxidants [35, 36]. However, approximately 30% of global fruit production is lost during the postharvest handling, distribution, and storage stages, largely due to fungal pathogens such as *B. cinerea*, *Penicillium* spp., and *Colletotrichum* spp. [3, 4, 37–39]. It is known that the plant defense systems in fruits are affected at the moment of detachment from the plant due to the interruption of the vascular flow of nutrients and chemical signals required to activate systemic defenses [40]. Consequently, harvested fruits rely on local defense mechanisms, including the production of phytoalexins, pathogenesis-related proteins (PRs), reactive oxygen species (ROS), and cell wall reinforcement. Nevertheless, such responses are often insufficient, leaving fruits highly vulnerable to pathogen attack as they undergo accelerated senescence [25, 41].

While chemical agents are widely used to control postharvest pathogens, their effectiveness is limited by negative side effects [42]. Therefore, biological control strategies using BCAs have gained increasing attention in recent years as a sustainable alternative [16, 43, 44]. BCAs are typically isolated from soil or plant organs such as flowers, fruits, or seeds [45–48]. Interestingly, animals, such as amphibians, harbor bacterial communities on their skin that protect them against fungal pathogens [29]. Moreover, bacteria isolated from amphibians’ skin have shown to protect plants against pathogens [49]. Previous studies demonstrated that C23F, C26G, and C32I bacteria isolated from amphibians inhibited the growth of *B. cinerea* in *Arabidopsis thaliana* and reduced disease development in blueberry fruits [30]. Genomic analysis revealed that strains C26G and C32I belong to the genus *Acinetobacter*, and C23F to the *Enterobacteriaceae* family [32, 50]. However, whether these frog skin–derived bacteria can antagonize other agronomically relevant phytopathogenic fungi remained unexplored.

Susilawati, Iwai [49] reported that three bacterial strains isolated from Japanese frogs inhibited phytopathogenic fungi, including *B. cinerea*, *Colletotrichum orbiculare*, *Fusarium oxysporum* f. sp. *lycopersici*, and *Pyricularia oryzae*. Similarly, we found that the bacteria C23F, C26G, and C32I display strong antifungal activity against a range of phytopathogenic fungi responsible for postharvest fruit diseases, such as *P. digitatum*, *P. italicum*, *A. alternata*, and *A. niger*. Comparable antagonistic effects have been reported for *Bacillus* spp., *Streptomyces* spp., and *Pseudomonas* spp. [51–54].

One of the main mechanisms employed by BCAs is antibiosis, involving the secretion of toxic compounds that inhibit pathogen development [55]. Analysis of bacterial filtrates from C23F, C26G, and C32I confirmed the presence of diffusible antifungal compounds that inhibited *B. cinerea*, *A. alternata*, *P. digitatum*, and *P. italicum*. Similar effects have been reported for *Pseudomonas aureofaciens* and *Bacillus subtilis C9*, which secrete compounds active against fungi such as *Pythium ultimum*, *Fusarium solani*, *F. oxysporum*, and *Rhizoctonia solani* [56, 57]. Additionally, VOCs released by these bacteria also contribute to fungal inhibition, as previously described in *Burkholderia tropica*, which affects the growth of *Colletotrichum gloeosporioides*, *Fusarium culmorum*, *F. oxysporum*, and *Sclerotium rolfsii* [58]. Together, these findings indicate that C23F, C26G, and C32I might suppress phytopathogenic fungi through at least two distinct mechanisms: the secretion of diffusible antifungal compounds and the emission of inhibitory VOCs [59].

With the goal of identifying the antifungal compounds produced by the BCAs, our previous comparative genomic analysis of strains C32I and C26G revealed biosynthetic gene clusters (BGCs) and chitin-degrading gene families (ChDGFs) differentially associated with the inhibition of fungal pathogens such as *B. cinerea* and the amphibian pathogen *B. dendrobatidis*. Specifically, these strains possess BGCs related to non-ribosomal peptide synthetases (NRPSs, including those involved in the production of non-alpha poly-amino acids (NAPAA), and ribosomally synthesized and post-translationally modified Peptides (RiPPs)). Both strains also harbor ChDGFs (GH18, GH19, and GH23) that are linked to their antifungal activity. The identified BGCs and ChDGFs may contribute to the synthesis of antifungal metabolites such as viscosin, fengycin, zwittermicin, various siderophores, and a novel family of β-lactones [60]. In contrast, for strain C23F, we do not yet have specific genomic information to identify the compounds potentially involved in its antifungal activity.

The efficacy of BCAs is influenced by the composition and structure of fungal cell walls. For instance, *G. citri-aurantii* has low chitin content and predominantly linear glucans, rendering it susceptible to bacterial lytic enzymes, although its rapid growth may partially counteract these effects [61, 62]. In contrast, *Fusarium* species modify their walls by increasing α−1,3-glucans, which mask β-glucans and reduce recognition by lytic enzymes and antifungal proteins [63]. *P. capsici*, with a cell wall rich in cellulose and β-glucans, is less affected by chitinase-based mechanisms but remains susceptible to other enzymes and bacterial secondary metabolites [64]. Conversely, *B. cinerea* and *A. alternata*, whose walls are rich in β−1,3-glucans and chitin, are effectively targeted by bacterial chitinases and glucanases [65, 66]. Overall, these findings highlight that cell wall composition not only governs fungal morphology and viability but also shapes interactions with BCAs, underscoring the importance of pathogen-specific structural characterization to optimize antifungal bacterial applications.

Finally, we demonstrated that cell suspensions and bacterial filtrates from C23F, C26G, and C32I are potential candidates to control *P. digitatum*, *B. cinerea*, and *A. alternata* in citrus, tomato, and blueberry, respectively. *Bacillus* spp. isolated from blueberry fruits and flowers have similarly reduced gray mold disease caused by *B. cinerea* [20]. Exogenous application of bacteria isolated from Japanese frogs on tomato plants significantly reduced disease development caused by *Fusarium oxysporum* f. sp. *lycopersici* [49]. Furthermore, inoculation of C23F, C26G, and C32I in the model plant *A. thaliana* activates defense mechanisms through the expression of genes associated with the jasmonic acid (JA), salicylic acid (SA), and ethylene (ET) signaling pathways, resulting in the inhibition of disease development caused by *B. cinerea* (31).

## Conclusion

In this study, we identified the antifungal potential of the bacterial strains C23F, C26G, and C32I, isolated from frog skin, against several economically important phytopathogenic fungi. These strains significantly inhibited the growth of *P. digitatum*, *P. italicum*, *B. cinerea*, *A. alternata*, *A. niger*, and *A. solani*. Moreover, in vivo assays demonstrated a marked reduction in disease incidence in citrus fruits, tomato, and blueberry fruits following the application of bacterial cell suspensions and filtrates, with the most notable protective effect observed against *P. digitatum* in lemon. These findings support the use of frog skin–derived bacteria as promising biological control agents with broad-spectrum antifungal activity. Their application could represent an effective and sustainable strategy for the management of postharvest fungal diseases in horticultural crops. However, further studies are required to characterize the bioactive compounds and evaluate their performance under commercial storage conditions.

## Supplementary Information

Below is the link to the electronic supplementary material.

ESM 1 (ZIP 3.62 MB)

## Data Availability

No datasets were generated or analysed during the current study.
