# Supplementary figures and images for: Bacteria from the Amphibian Skin Inhibit the Growth of Phytopathogenic Fungi and Control Postharvest Rots
Source: Microb Ecol. 2025 Sep 30;88(1):101. doi: 10.1007/s00248-025-02611-3 (PMC12484273; doi:10.1007/s00248-025-02611-3)

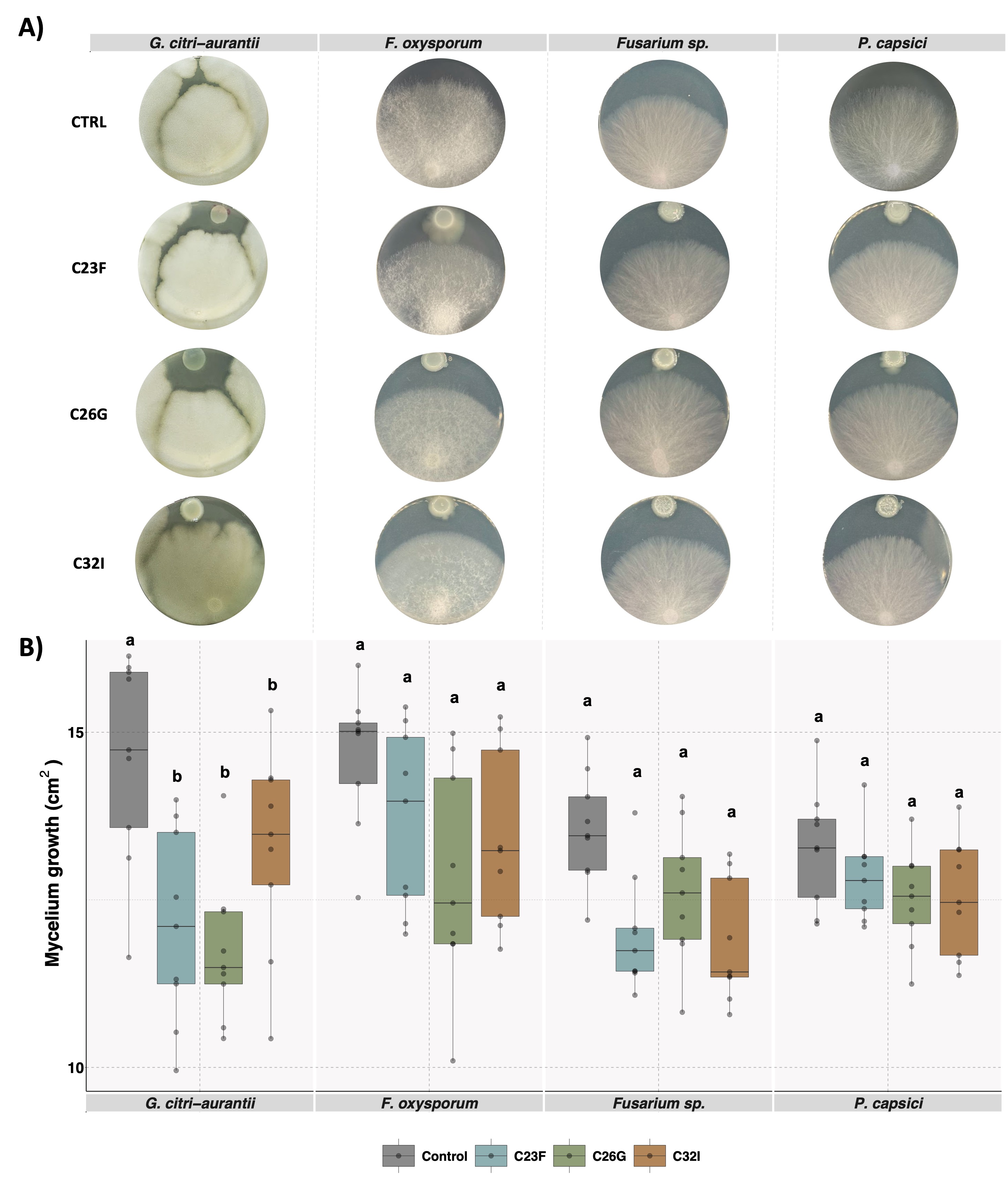

Supplement: Supplementary file 1 — (ZIP 3.62 MB) [file 248_2025_2611_MOESM1_ESM.zip › Supplementary__Figure_1.jpg]

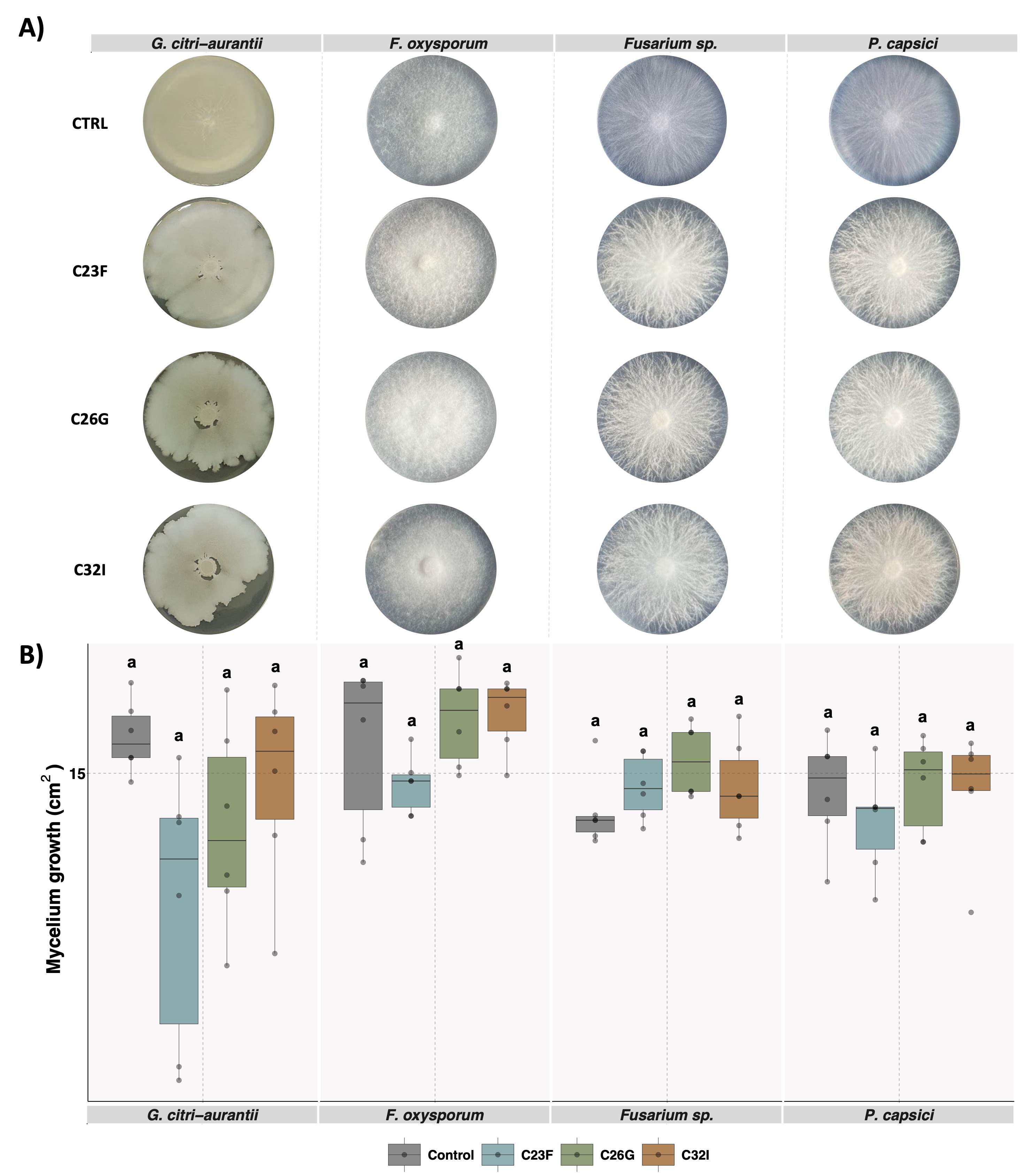

Supplement: Supplementary file 1 — (ZIP 3.62 MB) [file 248_2025_2611_MOESM1_ESM.zip › Supplementary__Figure_2.jpg]

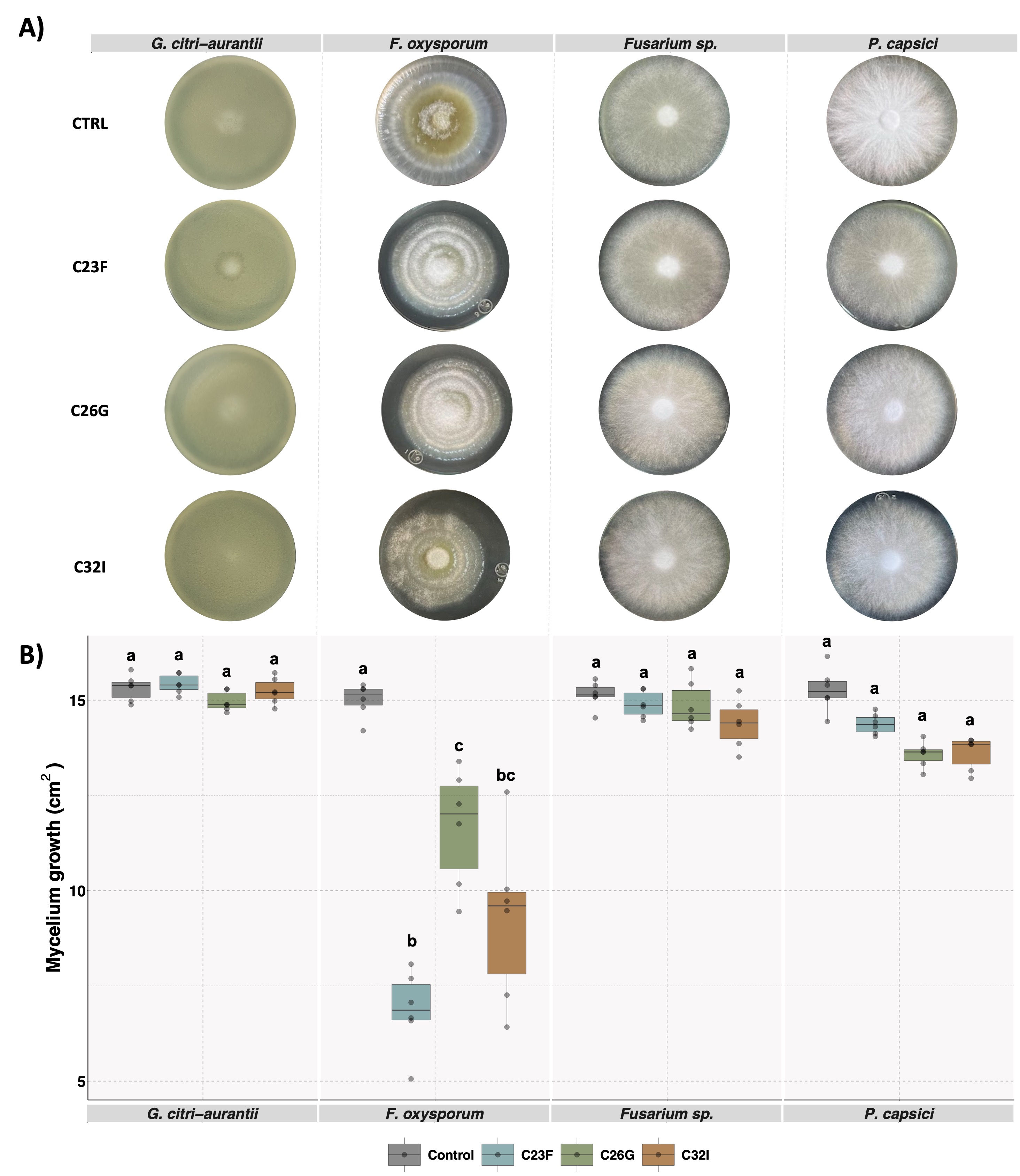

Supplement: Supplementary file 1 — (ZIP 3.62 MB) [file 248_2025_2611_MOESM1_ESM.zip › Supplementary__Figure_3.jpg]

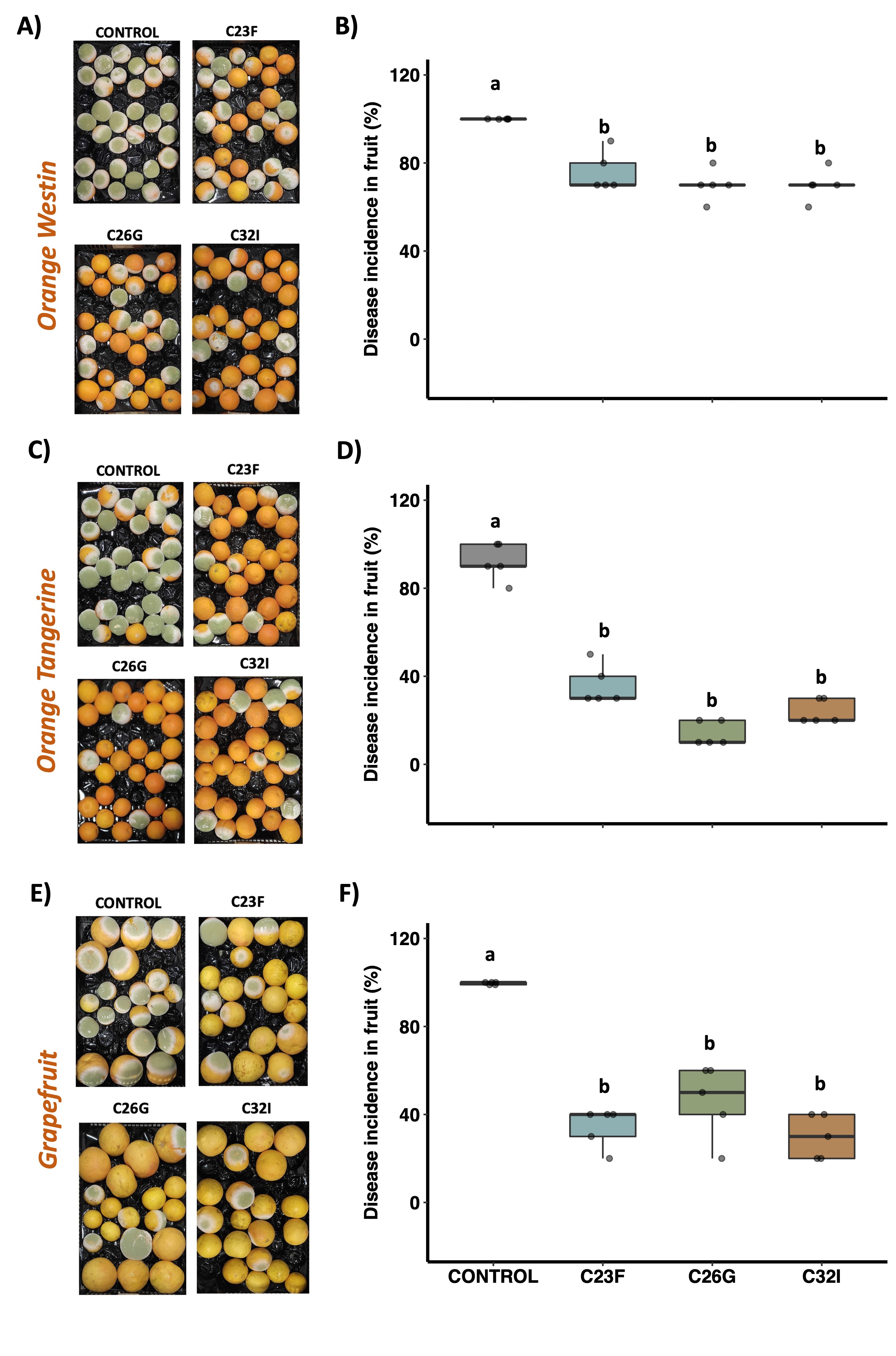

Supplement: Supplementary file 1 — (ZIP 3.62 MB) [file 248_2025_2611_MOESM1_ESM.zip › Supplementary__Figure_4.jpg]

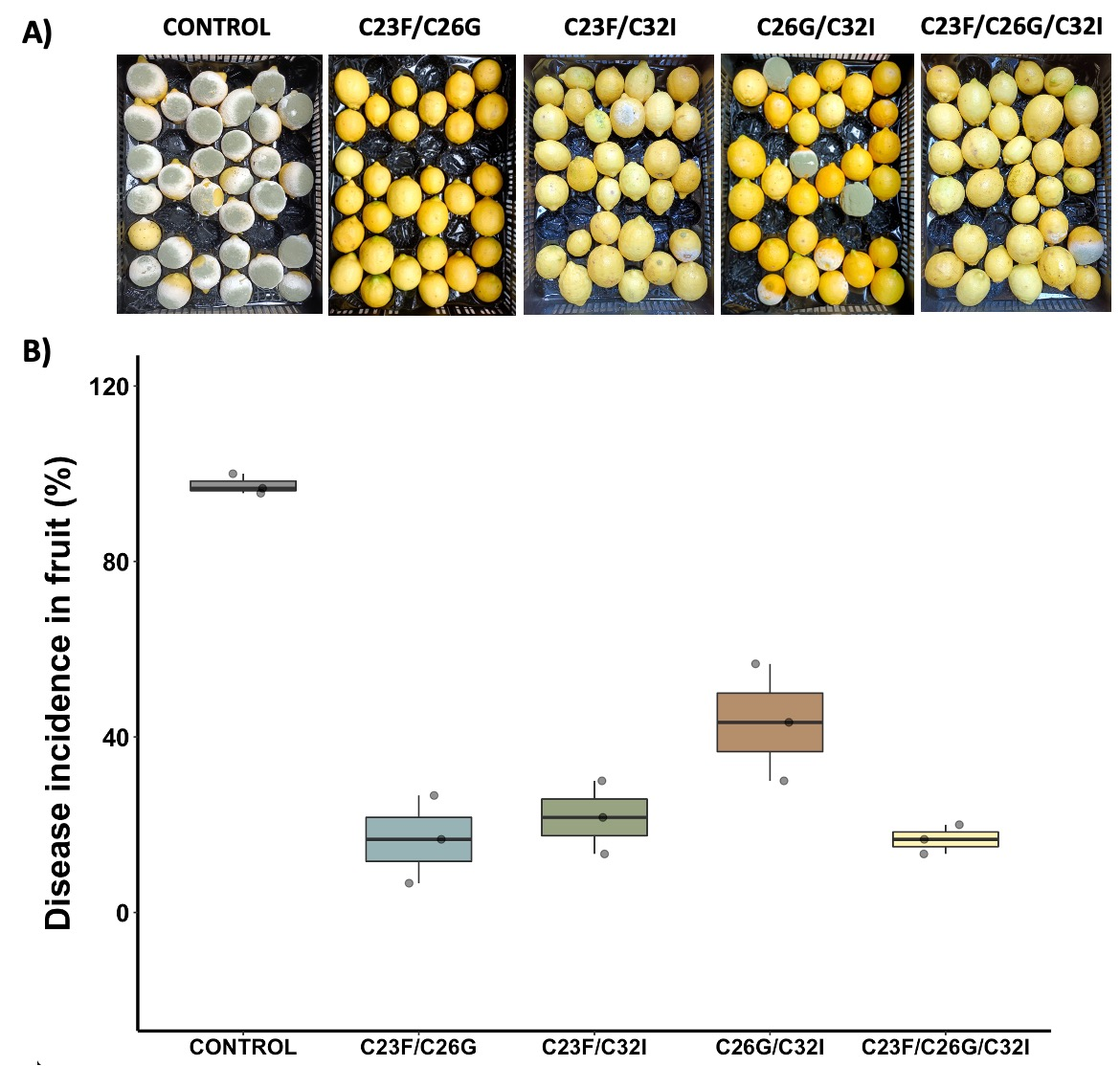

Supplement: Supplementary file 1 — (ZIP 3.62 MB) [file 248_2025_2611_MOESM1_ESM.zip › Supplementary__Figure_5.tiff]
